# Polymer Membranes of Zeolitic Imidazole Framework-8 with Sodium Alginate Synthesized from ZIF-8 and Their Application in Light Gas Separation

**DOI:** 10.3390/polym15041011

**Published:** 2023-02-17

**Authors:** Aftab Aslam Parwaz Khan, Mallikarjunagouda B. Patil, Laxmibai P. Rathod, Shivalila G. Vader, Pankaj Raizada, Pardeep Singh, Maha M. Alotaibi, Mohammad Omaish Ansari, Anish Khan, Naved Azum, Malik Abdul Rub, Muhammad Nadeem Arshad, Abdullah M. Asiri

**Affiliations:** 1 Center of Excellence for Advanced Materials Research, King Abdulaziz University, Jeddah 21589, Saudi Arabia; 2 Bharat Ratna Prof. CNR Rao Research Centre, Basaveshwar Science College, Bagalkot 587101, India; 3 School of Advanced Chemical Sciences, Shoolini University, Himachal Pradesh, Solan, 173229, India; 4 Chemistry Department, Faculty of Science, King Abdulaziz University, Jeddah 21589, Saudi Arabia; 5 Center of Nanotechnology, King Abdulaziz University, Jeddah 21589, Saudi Arabia

**Keywords:** polymer membrane, MOF, ZIF-8, gas permeation

## Abstract

The potential of nanocomposite membranes (NCMs) prepared by the sodium alginate polymer and embedded with synthesized zeolitic imidazole framework-8 (ZIF-8) as fillers having microporous structure in the application of separation of gaseous mixture generated by the process of methane reforming was assessed. ZIF-8 crystals were created through hydrothermal synthesis, with sizes varying from 50 to 70 nm. NCMs were prepared with a 15% filler loading, i.e., synthesized ZIF-8. NCMs (ZIF-8) having H_2_ permeability of 28 Barrer and H_2_/CH_4_ selectivity of 125 outperformed neat polymer membranes in terms of separation performance at ambient temperature and 4 kg/cm^2^ pressure. The purity of H_2_ increased to as high as 95% among the measured values. The NCMs did not, however, outperform a neat polymer membrane in terms of their ability to separate mixtures of gases. Moreover, the combination of ZIF-8 as a filler with sodium alginate was new and had not been reported previously. As a result, it is worthwhile to investigate.

## 1. Introduction

Carbonaceous gas reformation, in the same way as CO_2_ and CH_4_, is considered a promising strategy to combat global warming [1]. The results of reforming processes are beneficial for the production of chemicals and energy. Due to the chemical stability of both CO_2_ and CH_4_, it is difficult to reform all feed gases into usable products. Studies on catalyst design, engineering reforming reactions, and device development have been conducted to optimize specific reforming reactions [2]. Only a small portion of CO_2_ and CH_4_ gases are involved in the reactions in the majority of commercially viable reforming reactions; the remaining gases do not react and instead combine with the products. It is necessary to separate the reformate gases from the unreacted CO_2_ and CH_4_ if you intend to use them later.

Due to its low energy consumption and straightforward design, membrane-based gas separation is considered a potent substitute for traditional gas purification methods [3], like cryogenic distillation, absorption, and adsorption. These characteristics also facilitate simple hybridization with already-in-use reaction systems. Membrane separation is used to separate gas mixtures with different permeabilities due to diffusivity and solubility differences [4,5]. It is considered that the reforming reactions of CO_2_ and CH_4_ can produce hydrogen, ethylene, propane, acetylene, and benzene. Membrane-based processes are used to separate these reformates.

Polymers are proven to be the most common membrane constituents employed for gas separation today. In addition to treating natural gas, recovering hydrogen, and recovering vapor, polymer-based membrane separation systems have already been commercialized [6,7]. Swelling and polymer fractional free volume (FFV), which is an empirical value and dimensionless quantity which is the reason for the characterization of free volume in the polymers, on the other hand, significantly reduce the polymer membrane’s performance in separation and purification for a technique involving huge quantities of condensable gases in the feed gas or bulky gaseous molecules passing through the membrane [8]. Because such gas mixtures contain large and condensable gases as a result of CO_2_ and CH_4_ reformation, polymer membranes have difficulty separating them, necessitating research into next-generation membrane materials.

To address the shortcomings of polymeric membranes, researchers have investigated microporous membranes made of zeolites, carbon molecular sieves (CMS), and metal oxide framework (MOFs)-based films [9]. However, mass production of such membrane materials is a difficult problem. Mixed matrix membranes (MMMs), as opposed to polymer membranes, are scaled up without difficulty, provide exceptional separation concert, and have longstanding stability [10]. Microporous fillers like porous carbon, carbon nanotubes, zeolite, and MOFs are combined with polymers to create MMMs [11,12,13,14,15,16]. MOFs have received a lot of attention as fillers for NCMs and MMMs due to their tuneable pore structures and adaptable outlines, which contain exceptional compatibility with polymer matrixes. The resulting NCMs were investigated for gas separation methods such as CO_2_/N_2_, H_2_/CO_2_, CO_2_/C_3_H_8_, and H_2_/CH_4_ separation [17,18]. Using ZIF-8, a subfamily of MOFs, the polyimide was mixed with ZIF-8. The gas permeability and selectivity of the synthesized NCMs exceeded those of a neat polyimide membrane. They even exhibited molecular sieving features at advanced ZIF-8 loading (>50% *w*/*w*). For the manufacture of NCMs, it was used as an additive and mixed with polyether block amide, PIM-1, and polyimide [14,19,20]. The NCMs containing amine-functionalized UiO-66 displayed high crystallinity and CO_2_ affinity. Using co-polyimide/ZIF-8 NCMs, CO2/CH4 and propylene/propane separation were investigated [21,22].

There have been numerous studies conducted on nanomaterial-based membranes using MOFs and NCMs based on the permeation of a single gas through the membrane. The majority of these investigations have focused on CO_2_ separation. Following the reformation of carbonaceous gas, which frequently includes more than three gases, such as CO_2_ and CH_4_, sodium alginate is proven to be an excellent membrane-forming natural polymer and has several advantages, such as its membrane forming nature, flexibility, and good compatibility with nanoparticles. The main advantages of choosing ZIF-8 as a filler for the nanocomposite membranes are its molecular sieving effect, facile synthesis root, and excellent compatibility with the polymer matrix. The system of ZIF-8 nanoparticles as filler and sodium alginate as the base polymeric matrix has not been reported elsewhere. Hence, it is interesting to study the NCMs of ZIF-8 with sodium alginate, as it is new and interesting in the performance of light gas separation. It is important to investigate how well NCMs separate using multimolecular gas sources, as such, NCMs consisting of MOFs were created, and the gas separation capabilities for gases containing CH_4_, Ar, and H_2_, were investigated. In this study, sodium alginate with gas permeability was used, and based on these results, gas selectivity was calculated as a base polymer matrix along with ZIF-8, which is primarily used in NCMS research.

## 2. Materials and Methods

### 2.1. Crystallization of ZIF-8

Zn(NO_3_)_2_6H_2_O (663 mg, 1 mmol) and 2-methylimidazole (8 mmol, 304 mg) were combined in 22.4 mL of methanol in a glass vessel, and allowed to react at 65 °C for 12 h to create ZIF-8 [23]. After solvothermal synthesis, the products were centrifuged for purity before being cleaned three times in methanol to get rid of any unreacted substances. The products were then dried in an 80 °C oven for an entire night.

### 2.2. Mixed Membrane Preparation

The solution casting followed by solvent evaporation technique was adopted for the casting of both nascent Na-Alg and NCM membranes. The nascent Na-Alg membrane was prepared by dissolving 5 mg of Na-Alg polymer in 100 mL of deionized water, which made it homogeneous. Once the solution was a viscous slurry, it was then cast onto a neat and clean glass plate. Before starting the process, the laboratory-synthesized ZIF-8 NPs were vacuum dried for 10 h at 80 °C before being mixed with water and sonicated for 120 min. Then, arouse solution was used to dissolve commercial sodium alginate (Na-Alg). The ZIF-8/Na-Alg weight ratio in the water was 15% (*w*/*w*). Ten hours were spent vigorously stirring the solution at room temperature. In the following step, a spotless glass surface was cast with the solution and dried at 35 °C for 48 h. The cast films were then naturally cooled after being annealed for 24 h at 80 °C in a vacuum oven. Membranes ranging in thickness from 70 to 73 μm were measured with a digital micrometer screw gauge. This was supported by a SEM cross-sectional view. 

### 2.3. Characterizations

FTIR (Make: Shimadzu, Kyoto, Japan, Model: IR-Affinity-1) was used to determine the functional groups. A Siemens D5000 diffractometer was used for X-ray diffraction (XRD) analysis. X-rays were emitted towards the sample at room temperature and diffracted at different angles (2) and intensities by CuKα radiation with a wavelength of λ = 1.54. The surface and cross-sectional morphology of the prepared membranes were analyzed using scanning electron microscopy (SEM) (Make: JOEL, Model: JSM 840A) with a high magnification of 20,000× at 30 kV. ZIF-8 NPs and NCMs were photographed using a SEM. The membranes were placed on a copper stub, and then gold was sputtered onto them to make them conduct before the images were taken. Particle size measurements of the synthesized ZIF-8 nanoparticles were conducted using an Anton Paar particle size analyzer (model: PSA 1190)

Micromeritics Tristar 3020 was used to measure the N_2_ isotherm curves at liquid nitrogen temperature (77 K) (Micromeritics, Bedford, LU6 1AT, UK). In order to prepare for an analysis of N_2_ sorption, the degassing was carried out at 424 K for 10 h under a maintained vacuum (105 Torr). With P/P_0_ = 0.95, the pore volumes of the samples were determined based on the Brunauer–Emmett–Teller (BET) equation.

TGA (thermogravimetric analysis) was used to characterize the thermal properties of the sample (model: TGA 8000, make: Perkin Elmer, Waltham, MA, USA). The samples were kept in a chamber with an NH_4_Cl salt solution for 12 h prior to TGA, which was conducted in an environment with a relative humidity of 80%. As nitrogen flow was maintained (30 mL/min) and the temperature was increased at 5 °C per minute until 700 °C, a thermal analysis was performed.

### 2.4. Permeability Measurements

**Figure 1:**
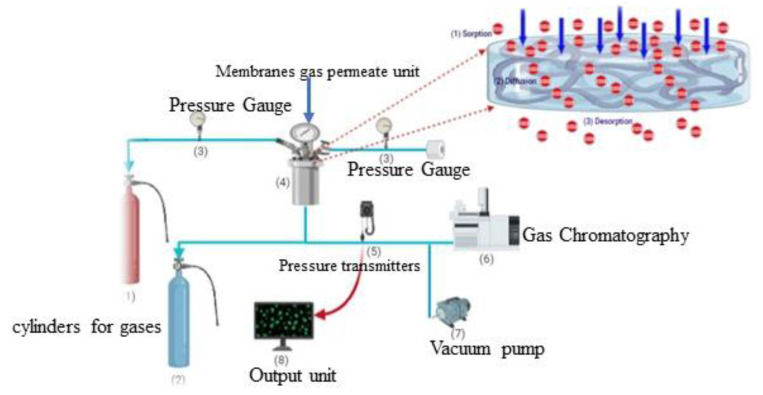
The setup for gas permeation is depicted in this diagram.

Using an indigenously built gas permeation unit and adopting a constant volume and variable pressure method, the NCMs’ single gas permeability was assessed. The membranes (9.4 cm^2^) were fixed on top of the gas permeation cell when the permeation pressure reached a steady state on the permeate side. Figure 1 shows the image. A gas permeameter was used to measure the permeabilities of N_2_ and CO_2_. Stainless steel permeation cells, which make up the permeameter (permeate side), separate upstream (feed side) and downstream (permeate side). A transducer measures the change in permeate pressure over time on the downstream side. A 13.302 cm^2^ area of the membrane is exposed to the gas in this cell. Pure gas permeation was measured using a constant volume variable pressure. An increase in pressure was plotted over time based on the raw data. The following equation can be used to calculate gas permeability:

(1) $$P=\frac{Vl}{A}\frac{T_{0}}{P_{f}P_{0}T}\left[{\left(\frac{dp}{dt}\right)}_{ss}-{\left(\frac{dp}{dt}\right)}_{leak}\right]$$

where:

- *P* is the permeability of the gas through the membrane (barrer);
- (1 Barrer = 10^−10^ cm^3^ (STP) cm cm^−2^ s^−1^ cmHg^−1^);
- *V* is the permeate volume (cm^3^);
- *l* is the thickness of the membrane layer (cm);
- *A* is the effective area of the membrane (cm^2^);
- P_f_ is the feed pressure (cmHg);
- P_0_ is the pressure at the standard state (76 cmHg);
- T is the absolute operating temperature (K);
- T_0_ is the temperature at the standard state (273.15 K);
- (dp/dt)ss is the steady-state pressure increase in the permeate side (cmHg s^-1^) under the feed pressure;
- (dp/dt)leak is the pressure increase in the permeate side under vacuum (leakage pressure increase).

Gas pairs A and B were calculated to determine their ideal selectivity, α*_A_*_/*B*_. The ratio of their permeability is defined as follows:

(2) $$\alpha_{A/B}=\frac{P_{A}}{P_{B}}\;.$$

## 3. Results and Discussion

### 3.1. Characterization of ZIF-8

**Figure 2:**
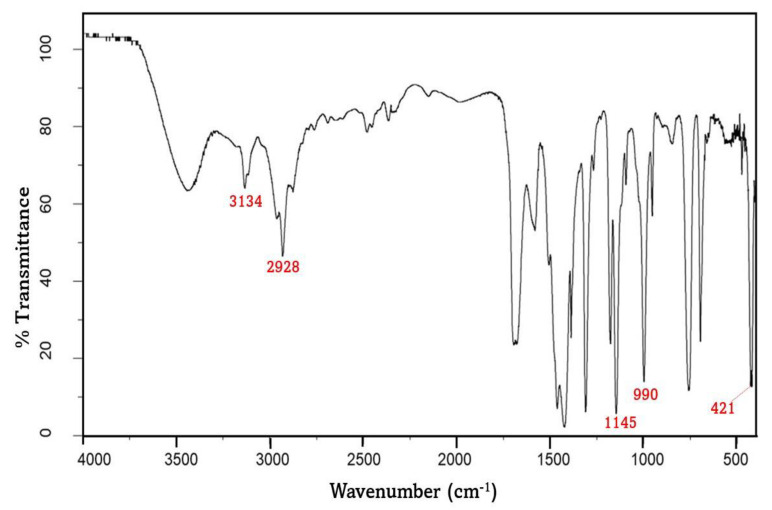
FTIR peaks of ZIF-8 nanoparticles.

Figure 2 depicts an FTIR analysis of ZIF-8 nanocrystals prepared in methanol. Clearly, the spectrum corresponds to the pattern described in the literature [23,24]. Zn-N stretching causes the absorption bands at 421 cm^−1^. The C-N absorption bands are associated with the peaks found between 900 and 1400 cm^−1^ (1145 and 990 cm^−1^). There are absorption bands at 2928 cm^−1^ and 3134 cm^−1^ associated with the aromatic and aliphatic C-H stretching of the imidazole, respectively.

### 3.2. XRD

**Figure 3:**
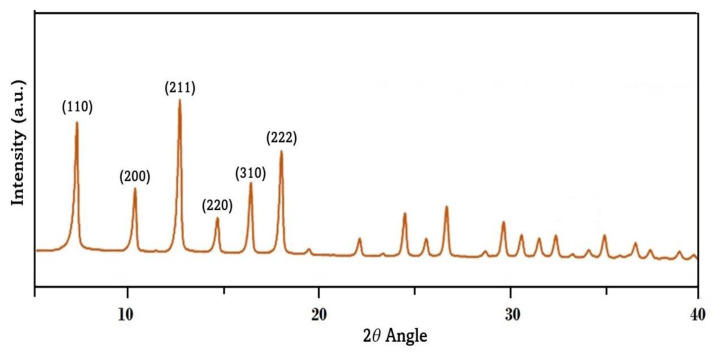
XRD pattern of synthesized ZIF-8.

The X-ray analysis for the synthesized ZIF-8 NPs is shown in Figure 3. The strong peaks in the 2*θ* angle = 7.30 which corresponds to the plane of (110), similarly, 10.34 (200), 12.7 (211), 14.81 (220), 16.42 (310), and 18.1 (222) planes. These peaks are the perfect structure of crystalline ZIF-8. 

### 3.3. SEM and Adsorption Analysis

**Figure 4:**
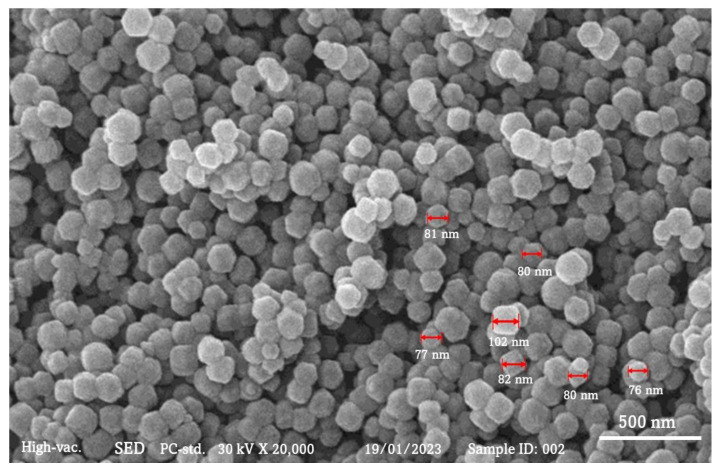
Scanning electron microscopy image of the zeolite imidazole framework (ZIF-8).

**Figure 5:**
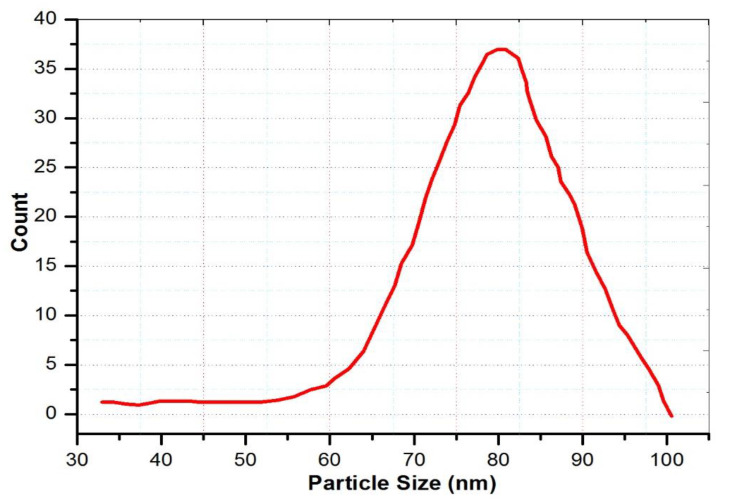
Particle size distribution histogram of zeolite imidazole framework (ZIF-8).

**Figure 6:**
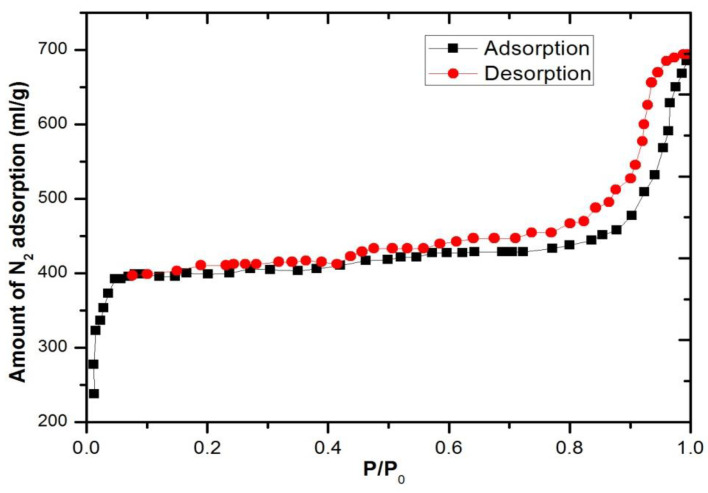
Nitrogen adsorption isotherm of ZIF-8 nanoparticles.

ZIF-8 crystals were morphologically and structurally confirmed using SEM and nitrogen isotherms. Figure 4 depicts the results of SEM micrograms. Figure 5 depicts the particle size of the ZIF-8, which ranged between 60 and 80 nm. According to ZIF-8 nanoparticle size analysis, this conclusion is supported. In Figure 5, we can see a histogram of particle size distribution. A type I isotherm was found for the nitrogen adsorption isotherm on ZIF-8, as shown in Figure 6. It is most likely that mesopores created by nanocrystal packing, which initiate nitrogen adsorption at 0.8 relative pressure, are responsible for the preliminary volumetric uptake of nitrogen gas at lower pressures. The BET surface area of ZIF-8 was found to be 1632 m^2^/g, and the total pore volume was found to be 1.09 cm^3^/g, which are comparable to literature values [25,26]. The obtained data from the measurement are shown in Table 1.

### 3.4. Characterization of Membranes

**Figure 7:**
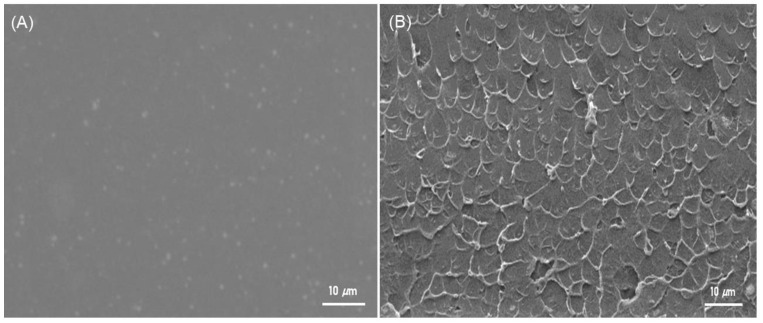
The images in (**A**) show plain Na-Alg membranes, and (**B**) have ZIF-8-filled NCMs.

**Figure 8:**
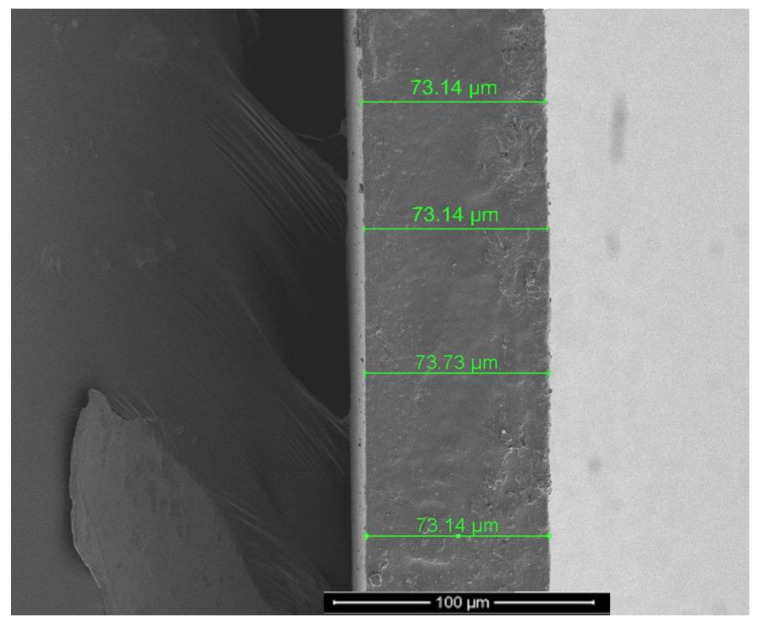
Cross-sectional image of NCMs (ZIF-8).

The synthesised ZIF-8 was used to prepare the NCMs at fixed loadings of 15 wt.%. Although more fillers could be incorporated into the polymer, a filler loading of 15% for NCMs (ZIF-8) was chosen after taking film processability and physical flexibility into account. As previously mentioned, a higher filler loading on NCMS resulted in improved gas permeability. The membrane, however, was a little more delicate and less reproducible. Additionally, a sizable membrane area (>15 cm^2^ in diameter) was necessary for this study’s gas flow meter and for the GC to detect detectable gas flux. Therefore, a reproducible and physically stable NCMS with a 15% filler loading was chosen. Figure 7A shows the plain Na-Alg, and Figure 7B displays an NCM’s (ZIF-8) SEM image. Folding the membrane in half did not cause it to break because it is flexible. According to a number of earlier studies, MOF nanoparticles did not disperse at elevated loadings, resulting in the development of morphologies resembling grapes [27]. However, ZIF-8 nanoparticle fillers were successfully incorporated into the polymer matrix with a 15 wt.% loading in the present investigation; Figure 7B illustrates this following the dispersion of particles in solvent with the aid of ultrasonication. This illustrates the favorable MOF and polymer interactions that have been published in earlier articles [28]. According to Figure 8, the NCMs had an average thickness of 73 µm. Therefore, it is believed that our approach enabled the successful preparation of NCMs.

**Figure 9:**
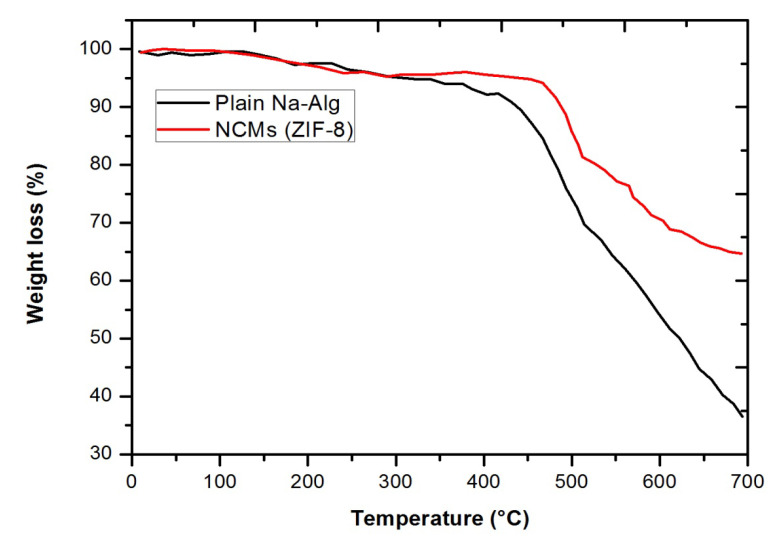
TGA curves of plain Na-Alg and NCMs (ZIF-8).

The thermal stability of the prepared samples is illustrated in Figure 9, including the plain Na-Alg polymer film and NCMs (15 wt.% ZIF-8 embedded Na-Alg membrane). Figure 9 demonstrates that below 200 °C, the ZIF-8 particles showed only minor losses as a result of trapped gaseous molecules and solvents. At 500 °C, the weight loss reached a plateau. Only 25% of the ZIF-8 was left after the TGA measurements because it decomposed at a temperature of about 400 °C. Continuous weight loss was noted between ~250 °C and ~500 °C due to polymer degradation. In addition, light gases such as hydrogen gas, carbon dioxide gas, and methane gas are evaporating from the polymer backbone [29,30]. The TGA results for NCMs (ZIF-8) revealed thermal decomposition characteristics linked to MOFs and polymer films, as shown in Figure 9. However, because ZIF-8 decomposition began earlier and overlapped with the temperature of polymeric film decomposition, the beneficial interaction with NCMS (ZIF-8) was not clearly revealed.

### 3.5. Single Gas Permeation through Membranes

**Figure 10:**
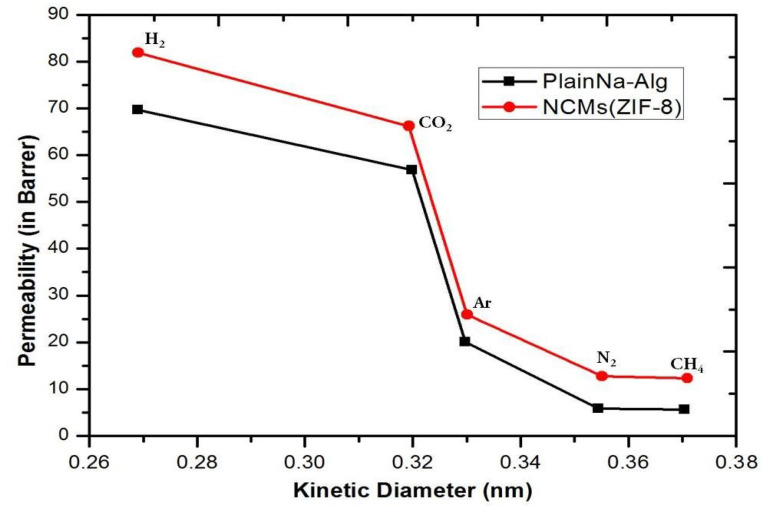
NCMs (ZIF-8) and plain Na-Alg membranes permeated by various gas molecules.

**Figure 11:**
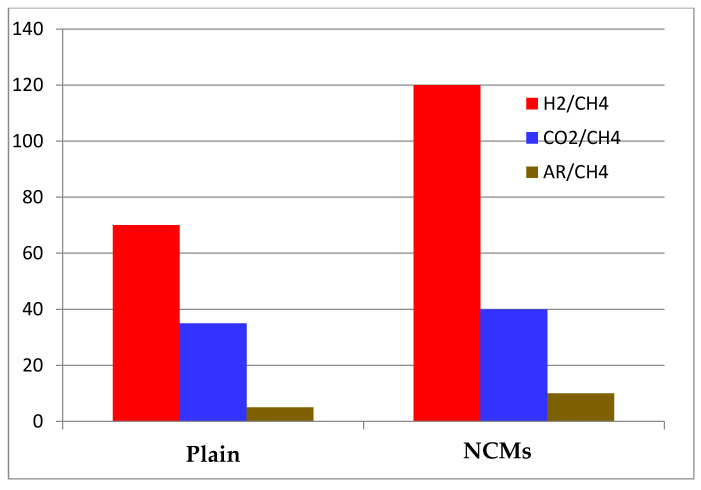
Na-Alg and NCMs’ (ZIF-8) membrane gas selectivity under various gases.

The results of single gas permeation for membranes made of pure polyimide and NCMs loaded with 15% ZIF-8 are shown in Figure 10. The gas separation experiment was conducted for the prepared membranes at 4 kg/cm^2^ pressure for a period of 2 hours. According to typical trends for gas separation membranes, as shown in Figure 10, the permeability of the gaseous molecules was reduced for all membranes with respect to the kinetically increasing diameter of gaseous molecules. A significant increase in gas selectivity and permeability was achieved by using MOFs in the polymer matrix (Figure 11). According to the literature, ZIF-8 is an effective filler for NCMs, and NCMs were effectively prepared without flaws. These increases in ideal gas selectivity and gas permeability prove that it is an effective filler for NCMs. Interestingly, despite ZIF-8’s pore size of approximately 0.35 nm, it is impermeable to CH_4_ and N_2_. NCMs (ZIF-8) have a higher gas permeability than Na-Alg. The “gate-opening” effect [31,32,33] of ZIF-8’s flexible structure appears to be more responsible for the increased permeability for N_2_ and CH_4_ than separation membrane flaws. [34,35]. The gas separation performance of the prepared NCMs (ZIF-8) was consistent, and they have been used three times to check the consistency and are stable with the same performance as the first time.

### 3.6. Comparison Study

The obtained results were compared with the literature’s reported values under the same operating conditions. The results were tabulated in Table 2.

It is observed from the collected data that the present work has much superior permeability values as compared with the reported literature values. 

## 4. Conclusions

The development of NCMs with MOFs as fillers was developed to investigate methane reforming by-product gas separation. The formation of ZIF-8 was confirmed using analytical characterization techniques such as SEM, XRD, and nitrogen adsorption isotherm measurement. It showed monodisperse crystal sizes and well-developed crystalline structures. Na-Alg and distinct MOF crystals were uniformly spread over a polymeric moiety due to the desired interactions between MOFs; SEM revealed no significant clusters or aggregates. The result of single gas permeation implies that many interactions occurred between diffusant gaseous molecules and diffusant-diffusant pairs. When prepared nanocomposite membranes are used, solution diffusion and surface pore diffusion can occur simultaneously, which may facilitate very complex gas diffusion paths. The membrane performance in terms of permeability and selectivity for the light gases H_2_, CO_2_, N_2_, CH_4_, and Ar were evaluated. The selectivity value for H_2_/CH_4_ was found to be the highest, and the smallest for Ar/CH_4_. In order for multicomponent separation via NCMs to be commercialized, extensive research is required.

## Figures and Tables

**Table 1:** The pore textural properties of virgin ZIF-8.

Sample	BET Surface Area (m^2^/g)	Mesopore Volume (cm^3^/g)	Micropore Volume (cm^3^/g)	Net Pore Volume (cm^3^/g)
ZIF-8	1632.3	0.521	0.569	1.09

**Table 2:** Comparison of the present work to the literature in terms of permeability.

Membrane	Operating Pressure (kg/cm_2_)	Temperature (°C)	PN_2_ (Barrer)	PCO_2_ (Barrer)	Reference
Hollow silica nanoparticles embedded hydroxyethyl cellulose membrane	4	30	10.10	71.30	17
Poly(vinyl alcohol)-g-starch methacrylate	4	30	0.674	10.474	18
ZIF-8/graphene oxide-15	4	30	0.31	14.50	35
Na-Alg-ZIF-8 (15 wt.%)	4	30	11.3	66.4	Present work

* P = permeability.

## Data Availability

The data presented in this study are available on request from the corresponding author.
